# Determinants of cancer mortality in patients after acute myocardial infarction

**DOI:** 10.1016/j.ijcha.2026.101914

**Published:** 2026-03-27

**Authors:** David Scharlach, Timo Schmitz, Philip Raake, Jakob Linseisen, Christa Meisinger

**Affiliations:** a Epidemiology, Medical Faculty, University of Augsburg, Augsburg, Germany; b University Hospital Augsburg, Department of Cardiology, Respiratory Medicine and Intensive Care, Augsburg, Germany

**Keywords:** Myocardial infarction, Cancer mortality, Lung cancer mortality, Gastrointestinal cancer mortality, Risk factors, Cardio-oncology

## Abstract

**Objective:**

To identify factors and preexisting conditions that are associated with overall cancer mortality, lung cancer mortality, and gastrointestinal cancer mortality in patients after acute myocardial infarction (AMI).

**Methods:**

In total 10,718 AMI patients aged 25 to 74 years were evaluated. All cases of AMI that occurred in the study region of the population-based Myocardial Infarction Registry Augsburg during the period from 2000 to 2017 were analyzed. Median follow-up time was 6.6 years (IQR 2.8–11.2). Multivariable Cox regression models were calculated for overall cancer mortality (ICD-10: C00-D48), lung cancer mortality (C34), and gastrointestinal cancer mortality (C15-C25).

**Results:**

During the study period, 633 patients died from cancer, including 155 deaths from lung cancer and 212 deaths from gastrointestinal cancer. Higher age, current smoking and diabetes were associated with an increased cancer mortality risk in patients after AMI, whereas female sex, never smoking and platelet aggregation inhibitor intake were inversely related to overall cancer mortality. Higher lung cancer mortality in patients after AMI was linked with advanced age and current smoking. For gastrointestinal cancer death in patients after AMI age, diabetes and current smoking were associated with higher risk, whereas female sex, never smoking, platelet aggregation inhibitor and statin intake were inversely associated.

**Conclusion:**

Factors already known for preventing cardiovascular disease were inversely associated with cancer mortality after AMI. These findings support the importance of comprehensive patient education regarding a healthy lifestyle following AMI. Overall, physicians targeting secondary prevention after AMI can also highlight the positive effects of these measures on cancer mortality.

## 1 Introduction

In the past decade, mortality due to acute myocardial infarction (AMI) has decreased in Europe [1]. The prognosis after a heart attack is influenced by a variety of factors, such as hypertension and diabetes, or other factors, like smoking, positive family history of cardiovascular disease (CVD) and older age [2], [3]. In contrast, the benefit of the evidence-based treatment after AMI, combining beta-blockers, aspirin/clopidogrel, statins and either angiotensin-converting enzyme inhibitors or angiotensin receptor blockers, for cardiovascular morbidity and mortality has been proven [4]. However, the extent to which these factors also affect other causes of death after AMI remains largely unexplored. This question is important, as recent studies observed an association between myocardial infarction and cancer incidence [5], [6]. Moreover, previous research suggested that the cancer mortality in patients after AMI is increasing [7]. Hence, the objective of this study was to identify factors and preexisting conditions after AMI that are associated with overall cancer mortality. Furthermore, this study searched for risk and protective factors, specifically for lung cancer mortality and gastrointestinal cancer mortality, in patients who suffered from AMI.

## 2 Materials and methods

### 2.1 Study population

For this analysis, data were obtained from the population-based Augsburg Myocardial Infarction Registry. Until 1995, this registry belonged to the WHO Monitoring Trends and Determinants on Cardiovascular Diseases (MONICA) project. It subsequently operated as KORA Myocardial Infarction Registry and has been transitioned into the Augsburg Myocardial Infarction Registry in 2021. The registry records patients with AMI whose primary residence is in the city of Augsburg, Germany, or the surrounding counties of Augsburg and Aichach-Friedberg, covering a total population of approximately 680,000 people in the study area. Until 2008, the registry only recorded patients aged 25 to 74 years, afterwards AMI patients from 25 to 84 years have been included. The registry collects data during the hospital stay, such as sociodemographic characteristics, acute symptoms, risk factors, comorbidities, diagnostics, and treatment, by conducting standardized interviews and elaborating medical files. After discharge, the registry regularly surveys the regional registration and health care offices to obtain information on survival of the AMI patients. In the event of a patient's death, death certificates are obtained from local health departments, and the underlying cause of death was coded by a single trained employee using the tenth revision of the International Classification of Diseases (ICD-10). The final date of mortality follow-up in the data set was June 30, 2019. The ethics committee of the Bavarian Medical Association (Bayerische Landesärztekammer) approved the study (ethics vote number 12057), which was performed in accordance with the principles of the Declaration of Helsinki. All the participants provided written informed consent. Further information about the Augsburg Myocardial Infarction Registry can be found in previous publications [8], [9], [10].

### 2.2 Outcome and study sample

The outcomes were categorized using the ICD-10 code as follows: overall cancer death (C00-D48), lung cancer death (C34) and gastrointestinal cancer death (C15-C25). This study considered patients aged 25 to 74 years, who suffered from AMI between January 1, 2000, and December 31, 2017. A total of 10,718 patients were included in the final analysis.

### 2.3 Statistical analysis

Categorial variables are presented as total numbers and percentages and continuous variables are shown as either medians and interquartile ranges (IQR) or means and standard deviations (SD). To examine the associations between various factors or pre-existing conditions and cancer mortality, Kaplan-Meier survival curves and multivariable Cox regression models were conducted. Patients with any prevalent cancer at baseline were excluded from the Kaplan-Meier and Cox regression analyses for all three outcomes.

For the outcomes overall cancer death, lung cancer death and gastrointestinal cancer death, Kaplan-Meier curves were stratified by the following potential predictors: age (quartiles), sex, BMI (quartiles), type of infarction, therapy with PCI, hypertension, diabetes, smoking status (current smoker, ex-smoker, never smoker), level of education, platelet aggregation inhibitor (PAI) use at discharge, and statin intake at discharge. Log-rank tests were used to assess differences in survival between the groups. For each of the three outcomes, variables with significant log-rank tests were included in a subsequent multivariable Cox regression model. The proportional hazards assumption for the Cox models was checked graphically or by including a time-interaction term. For significant variables, E-values for the point estimates (HR) and the lower bounds of the 95% Cis were calculated to assess the strength of association a set of unmeasured confounders must have to nullify the observed significant association between exposure and outcome.

Several sensitivity analyses were performed. First, Cox regression models were conducted using only patients who survived at least two years after the acute event. Second, the three models from the main analysis were recalculated as Fine-Gray models to assess whether competing risk issues affected the associations observed in the cause-specific Cox regression models, as recommended by Ameri et al. in 2025 [11]. Third, to account for possible changes in AMI/post-AMI care during the study period, a sensitivity analysis was performed for the associations between exposures and overall cancer mortality including only patients with AMI between 2010 and 2017.

The statistical analysis was performed using SPSS statistics, version 29.0.1.0 and R version 4.4.3, and the significance level was set at p-value < 0.05.

## 3 Results

Table 1 shows the baseline characteristics of the study sample. A total of 10,718 AMI patients were included in the analysis, consisting of 8,095 men (75.5%) and 2,623 women (24.5%). At time of discharge, cancer was prevalent in 1,164 patients (10.9%), including 816 men and 348 women. Specifically, lung cancer was present in 48 patients (0.4%), and gastrointestinal cancer was prevalent in 155 patients (1.4%). A total of 644 patients died from cancer during the observation period, including 155 lung cancer deaths and 212 gastrointestinal cancer deaths.

**Table 1:** Baseline characteristics of AMI patients: categorial variables are displayed as total numbers (n, %). Numeric data is presented as mean (SD) or median (IQR).

	**total sample: n = 10718**	**n**
Female	2623 (24.5)	10,718
Age at infarction (mean, SD)	61.1 (9.6)	10,718
mortality	3637 (33.9)	10,718
28-days-mortality	644 (6.0)	10,718
cancer mortality (C00-D48)	633 (5.9)	10,718
lung cancer mortality (C34)	155 (1.4)	10,718
gastrointestinal cancer mortality (C15-C25)	212 (2.0)	10,718
prevalent cancer at discharge	1164 (10.9)	10,718
prevalent lung cancer at discharge	48 (0.4)	10,718
prevalent gastrointestinal cancer at discharge	155 (1.4)	10,718
Type of infarction:		10,278
STEMI	4001 (37.3)	
NSTEMI	5573 (52.0)	
Bundle branch block	704 (6.6)	
not defined	440 (4.1)	
Treatment:		10,702
CABG	1609 (15.0)	
PCI	7298 (68.1)	
Prehospital time in minutes	155.00 (82.00–486.00)	10,718
BMI in kg/mm^2^ (mean, SD)	27.8 (4.8)	9868
Hypertension	8027 (74.9)	10,707
Diabetes mellitus	3329 (31.1)	10,708
previous infarction:		10,703
yes	996 (9.3)	
no	9707 (90.6)	
no information	15 (0.1)	
previous stroke:		10,718
yes	766 (7.1)	
no	9225 (86.1)	
no information	727 (6.8)	
Smoking status:		9653
currently smoking	3850 (35.9)	
ex-smoker	3116 (29.1)	
never-smoker	2687 (25.1)	
no information	1065 (9.9)	
LVEF:		8032
<= 30%	573 (5.3)	
> 30%	7459 (69.6)	
no information	2686 (25.1)	
Highest school or university degree:		8551
lower secondary school“	5807 (54.2)	
higher educational attainment	2744 (25.6)	
no information	2167 (20.2)	
Laboratory value:		
Admission Troponin I (ng/ml)	0.58 (0.11–3.75)	10,718
peak CKMB levels (U/l)	53.00 (22.00–141.00)	10,718
Admission CRP (mg/dl)	0.47 (0.24–1.35)	10,718
Peak Glucose (mg/dl)	149 (122.00–204.00)	10,718
Medication at discharge:		
Anticoagulants	1335 (12.5)	9889
Platelet aggregation inhibitors	9589 (89.5)	9891
Beta blockers	9357 (87.3)	9892
Angiotensin-II antagonists	771 (7.2)	9886
ACE inhibitors	7572 (70.6)	9890
Calcium antagonists	1525 (14.2)	9886
Diuretics	4544 (42.4)	9887
Statins	8866 (82.7)	9889
Oral antidiabetics	1365 (12.7)	9886
Insulin	1016 (9.5)	9887

**Fig. 1:**
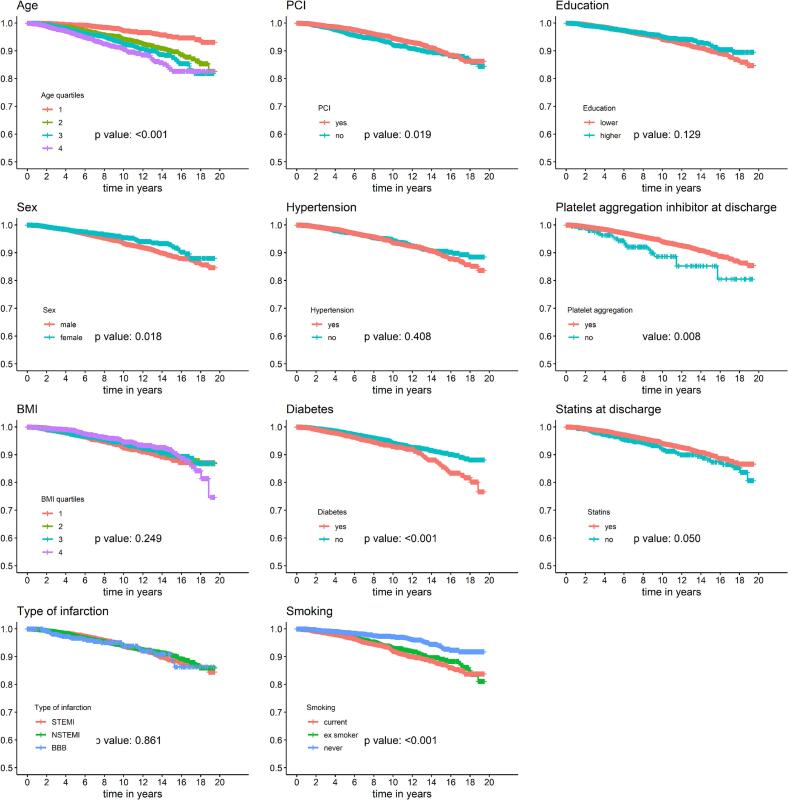
Kaplan Meier curves for the event cancer death in AMI patients, stratified by all initially considered variables; p-values were calculated by log-rank tests.

Fig. 1 provides Kaplan-Meier curves for overall cancer mortality in AMI patients. The Kaplan-Meier curves were startified by the preselected potential predictors mentioned above. The variables age, sex, PCI, diabetes, smoking, platelet aggregation inhibitors at discharge, and statins at discharge had significant log-rank test. For lung cancer, only age, smoking and education showed significant differences, see supplementary figure S1. For gastrointestinal cancer, age, sex, PCI, hypertension, diabetes, smoking, platelet aggregation inhibitors at discharge and statins at discharge reached significance in the log-rank test, see supplementary figure S2.

Table 2 displays the results of the Cox regression model for overall cancer death in AMI patients. Each additional year of age at infarction was associated with an 8% increase in overall cancer mortality risk. Female sex was associated with an approximately 30% risk reduction, while diabetes was associated with an increased risk of 37%. Current smokers had more than twice the risk of overall cancer mortality compared to ex-smokers, whereas never-smokers had approximately half the risk of ex-smokers. Patients who received a PAI at discharge had a lower overall cancer mortality risk compared to those who were not prescribed this medication.

**Table 2:** Hazard ratios (HR) and 95% confidence intervals (95%-CI) of the Cox regression analysis for the outcome cancer death in AMI patients. The models included the following variables: age, sex, PCI, diabetes, smoking status, platelet aggregation inhibitor at discharge and statin at discharge.

	**HR**	**95%-CI**	**p-value**	**E-value** **(lower 95%CI)**
Age	1.08	1.07–1.10	<0.001	1.37 (1.34)
Female sex	0.70	0.55–0.91	0.006	2.21 (1.43)
Therapy: PCI	0.94	0.76–1.17	0.579	
Diabetes	1.37	1.11–1.67	0.003	2.08 (1.46)
Smoking status: (ex-smoker)				
currently smoking	2.14	1.70–2.68	<0.001	3.70 (2.79)
never-smoker	0.49	0.37–0.66	<0.001	3.50 (2.40)
Platelet aggregation inhibitor at discharge	0.54	0.33–0.89	0.017	3.11 (1.50)
Statin at discharge	0.86	0.63–1.18	0.358	

Table 3 presents the results of the Cox regression model for lung cancer death. An advanced age at AMI was associated with an increased lung cancer mortality. Current smokers had a higher risk of lung cancer mortality compared to ex-smokers, while never smokers had a substantially reduced risk.

**Table 3:** Hazard ratios (HR) and 95% confidence intervals (95%-CI) of the Cox regression analysis for the outcome lung cancer death in AMI patients. The models included the following variables: age, smoking status, and education.

	**HR**	**95%-CI**	**p-value**	**E-value** **(lower 95%CI)**
Age	1.10	1.07–1.13	<0.001	1.43 (1.34)
Smoking status: (ex-smoker)				
currently smoking	4.51	2.78–7.34	<0.001	8.49 (5.00)
never-smoker	0.15	0.05–0.43	<0.001	12.81 (4.08)
Higher educational attainment	0.66	0.40–1.09	0.106	

Table 4 presents the results of the Cox regression model for the outcome gastrointestinal cancer death. As with overall cancer and lung cancer, higher age at infarction significantly increased the risk of dying from gastrointestinal cancer. Female patients had a significantly reduced risk, while patients with diabetes had a significantly higher risk of gastrointestinal cancer death. Moreover, never smokers had a reduced risk for gastrointestinal cancer death compared to ex-smokers. Finally, both PAI use at discharge and statin use at discharge were associated with a lower risk of gastrointestinal cancer death.

**Table 4:** Hazard ratios (HR) and 95% confidence intervals (95%-CI) of the Cox regression analysis for the outcome gastrointestinal cancer death in AMI patients. The models included the following variables: age, sex, PCI, hypertension, diabetes, smoking status, platelet aggregation inhibitor at discharge and statin at discharge.

	**HR**	**95%-CI**	**p-value**	**E-value** **(lower 95%CI)**
Age	1.07	1.05–1.09	<0.001	1.34 (1.28)
Female sex	0.61	0.39–0.96	0.033	1.25 (2.66)
Therapy: PCI	0.83	0.58–1.20	0.325	
Hypertension	1.23	0.80–1.89	0.337	
Diabetes	1.81	1.30–2.53	<0.001	3.02 (1.92)
Smoking status: (ex-smoker)				
currently smoking	1.47	1.00–2.16	0.052	
never-smoker	0.51	0.32–0.81	0.004	3.33 (1.77)
Platelet aggregation inhibitor at discharge	0.43	0.21–0.87	0.019	4.08 (1.56)
Statin at discharge	0.54	0.35–0.85	0.008	3.11 (1.63)

### 3.1 Sensitivity analyses

Including only patients who survived the acute event at least two years revealed similar associations to those found in the main Cox regression models (supplementary table S1, S2, S3). Furthermore, when the analysis was restricted to patients with AMI between 2010 and 2017, the results for overall cancer mortality were quite similar to the main analysis (supplementary table S7). Finally, the Fine-Gray models (supplementary table S4, S5, S6) largely confirmed the result of the main models, indicating that competing risk was not a major issue in the present analysis.

## 4 Discussion

In the present study, age, sex, diabetes, and smoking status were identified as main determinants of overall cancer mortality after AMI. The importance of smoking cessation after AMI should be emphasized, as it is also relevant for reducing lung cancer and gastrointestinal cancer mortality. Use of PAIs was associated with lower overall and gastrointestinal cancer mortality, and statin intake was associated with a lower risk of gastrointestinal cancer mortality. In addition, diabetes and sex were associated with gastrointestinal cancer mortality post-AMI.

### 4.1 Age

The overall risk of developing cancer in the general population increases with age, peaking at around 70 to 90 years depending on the type of cancer, after which it slightly declines [12], [13], [14]. Tamási et al. stated that lung cancer incidence and mortality reach their highest level between 70 and 79 years in male patients and between 60 and 69 in female patients [15]. Moreover, the risk of gastrointestinal cancer increases with age [16]. Since the average age of AMI patients in our study was about 61 years, this could explain the significantly increasing risk per year of age for all considered outcomes.

### 4.2 Sex

The International Agency for Research on Cancer published a global cancer statistic in 2022: it showed that for most cancer sites, men have both, higher incidence rates and higher mortality rates. Especially in the case of lung cancer males represent more than 60% of patients with newly diagnosed lung cancer [17]. A Swedish cohort study from 2017 found similar results [18].

### 4.3 Smoking

It is well known that smoking increases the risk of cancer and cancer mortality in the general population. A recent Australian population-based cohort study detected that current smokers had more than three times the risk of overall cancer death compared to never-smokers (HR: 3.23; 95%-CI: 2.94–3.55; p-value: <0.001) [19]. Lotan et al. displayed that smoking cessation after AMI reduces cancer risk [20]. We found that smoking in post AMI patients increases the mortality risk for all outcomes analyzed in the present study, but most dramatically for lung cancer mortality, which is equivalent to the general population [19].

### 4.4 Platelet aggregation inhibitors

Acetylsalicylic acid (ASA) significantly reduces mortality after AMI and is used for secondary prevention [21], [22], [23]. The scientific evidence regarding the reduction of cancer risk and mortality by the use of PAIs in the general population is fairly clear. A Spanish retrospective cohort study from 2023 reported that daily aspirin intake (between 75 mg and 250 mg) was associated with a reduced incidence of colorectal (HR: 0.70) and pancreatic cancers (HR: 0.50) [24]. García Rodríguez et al. found a 54% decrease in stomach cancer risk and a 41% reduction in esophageal cancer risk [25]. Moreover, a Danish cohort study determined significant risk reductions for some cancer sites including cancers of the colon (HR: 0.90), stomach (HR: 0.89), liver (HR: 0.90), pancreas (HR: 0.90) and small intestine (HR: 0.61) [26]. A *meta*-analysis from 2019 concluded that post-cancer diagnosis use of low dose ASA was associated with a reduction in cancer-specific mortality for some gastrointestinal cancers, including colorectal, esophageal and gastric cancers, but these associations were not found for pre-diagnosis low dose ASA intake [27].

### 4.5 Statins

Statins are established as part of secondary prevention after AMI [28], [29]. The data on the benefit of statins regarding the risk and mortality of gastrointestinal cancer in the general population is inconclusive. Different studies found that statins reduced the risk of pancreatic cancer in the general population [30] and lowered mortality risk in pancreatic cancer patients [31]. Other investigations found no link between statin use and pancreatic cancer risk [32], [33], [34] or pancreatic cancer mortality [33]. Regarding the risk and mortality of colorectal cancer (CRC) the data is controversial, too. While some studies suggested positive effects of statin use on CRC risk [35] and mortality [36], [37], others did not [38]. However, the present study found a significant risk reduction of gastrointestinal cancer mortality in statin users after AMI. Ren et al. reported similar results in patients with heart failure, where a lower risk of cancer and cancer-related mortality was observed in statin users [39]. Overall, statin use after AMI might be associated with a lower risk of gastrointestinal cancer death, in addition to its secondary preventive effects on the cardiovascular system.

### 4.6 Diabetes

Different studies and a *meta*-analysis from 2013 showed that there is a link between diabetes and cancer mortality in the general population [40], [41], [42]. In 2025 Storman et al. stated that type 2 diabetes is increasingly recognized as a risk factor for various gastrointestinal cancers due to chronic hyperglycemia, inflammatory processes, and gut microbiota dysbiosis [43]. Therefore, AMI patients with diabetes could be particularly vulnerable to cancer.

### 4.7 Hypertension

Two recent *meta*-analyses found that there is an association between hypertension and the risk for some cancer sites in the general population [44], [45]. The present study however revealed no association in patients after AMI. One possible explanation could be that hypertension solely increases the risk of certain rare cancers such as kidney cancer [44], for which no subgroup analyses could be carried out and which only account for a small proportion of overall cancer mortality. Another reason could be the fact that the blood pressure of post-AMI patients is monitored better and adjusted more precisely than in hypertensive subjects of the general population.

### 4.8 Possible mechanisms linking AMI and cancer mortality

Increasing evidence suggests a complex and bidirectional relationship between cardiovascular disease and cancer. Beyond shared lifestyle risk factors, emerging data indicate that biological alterations following AMI may directly influence cancer development and progression [46], [47], [48]. One proposed mechanism involves persistent systemic inflammation following myocardial injury. Necrosis of cardiomyocytes triggers a strong inflammatory response characterized by cytokine release, leukocyte recruitment, and activation of innate immune pathways [49]. While this inflammatory cascade is essential for tissue repair and cardiac remodeling, sustained systemic inflammation may create a pro-tumorigenic environment by promoting cellular proliferation, angiogenesis, and genomic instability [50], [51]. Closely related to this inflammatory state is immune system reprogramming after AMI [49]. Experimental studies have demonstrated that AMI can induce alterations in hematopoiesis and immune cell composition, particularly affecting monocyte and macrophage populations [52]. Such changes may impair anti-tumor immune surveillance and thereby facilitate tumor development and progression [53]. In addition, Clonal Hematopoiesis of Indeterminate Potential (CHIP) – a condition characterized by the age-related expansion of hematopoietic stem cell clones carrying somatic mutations, often in genes regulating epigenetic modification and inflammatory signaling – has emerged as a potential shared biological link between cardiovascular disease and cancer [54]. These mutations have been associated with increased cardiovascular risk through enhanced inflammatory activity and have also been linked to an elevated risk of hematologic malignancies, suggesting a common inflammatory and genetic substrate underlying both diseases [55]. Finally, recent experimental evidence indicates that the injured heart itself may release cardiac-derived circulating factors capable of influencing tumor biology [56]. In animal models, cardiac injury and subsequent heart failure have been shown to accelerate tumor growth through circulating mediators that promote tumor cell proliferation and survival [57]. Collectively, these mechanisms highlight the complex interplay between cardiovascular injury and oncologic processes and underscore the emerging field of cardio-oncology.

## 5 Strengths and limitations

The study was conducted using data from a population-based registry, resulting in a large number of AMI patients with consecutive enrollment and a long follow-up period, which reduces the risk of selection bias. Furthermore, comprehensive information for each patient was collected in a standardized manner, including socio-demographic information, treatment details, comorbidities, and life-style factors, enabling the inclusion of several factors in multivariable Cox regression models.

Nevertheless, there are some limitations as well. The findings may not be generalizable to older populations or all ethnicities because only patients from 25 to 74 years were considered and no data on patients’ ethnicities was collected. Complete case analyses were performed in the present study. Multiple imputation was not performed because missing values occured more frequently among more severely ill patients, suggesting a not-missing-at-random (MNAR) mechanism that violates the missing-at-random (MAR) assumption underlying standard multiple imputation methods. Another potential limitation is residual confounding. Patients with contraindications to antiplatelet therapy (e.g., gastrointestinal ulcers or severe liver disease) may have different baseline cancer risks, and detailed information on such contraindications was not available. Key covariates such as atrial fibrillation, anemia, and renal function were not available in our dataset. These factors may influence medication prescription patterns and could also contribute to residual confounding. Moreover, as medication data were restricted to discharge prescriptions, we were unable to account for changes in therapy or adherence during follow-up. Additionally, information on cancer treatment before or after AMI was not available, limiting interpretation of the observed associations. Also, the possibility of reverse causality cannot be ruled out for certain modifiable risk factors, such as comorbidities or medication use, where preclinical cancer may have influenced their presence or measurement at baseline. Moreover, immortal time bias cannot be excluded. Finally, we might not have considered all relevant factors in the multivariable models.

## 6 Conclusions

In this study, we identified several determinants of overall, lung and gastrointestinal cancer mortality in patients after AMI. Our findings demonstrated that factors known to prevent cardiovascular disease are associated with reduced cancer mortality after AMI. These results support the importance of comprehensive patient education regarding a healthy lifestyle following AMI. Physicians involved in secondary prevention for AMI patients may also refer to the potential associations of these measures with cancer mortality.

## CRediT authorship contribution statement

**David Scharlach:** Writing – original draft, Formal analysis, Conceptualization. **Timo Schmitz:** Writing – review & editing, Supervision, Methodology, Funding acquisition, Conceptualization. **Philip Raake:** Writing – review & editing. **Jakob Linseisen:** Writing – review & editing, Funding acquisition. **Christa Meisinger:** Writing – review & editing, Supervision, Methodology, Funding acquisition, Conceptualization.

## Declaration of competing interest

The authors declare that they have no known competing financial interests or personal relationships that could have appeared to influence the work reported in this paper.
