# Supplementary material for: Determinants of cancer mortality in patients after acute myocardial infarction
Source: Int J Cardiol Heart Vasc. 2026 Mar 27;64:101914. doi: 10.1016/j.ijcha.2026.101914 (PMC13059012; doi:10.1016/j.ijcha.2026.101914)
Supplement: Supplementary Data 1 [file mmc1.docx]

**Determinants of cancer mortality in patients after acute myocardial infarction**

David Scharlach, Timo Schmitz, Philip Raake, Jakob Linseisen, Christa Meisinger

Table S1: Hazard ratios (HR) and 95% confidence intervals (95%-CI) of the Cox regression analysis for the outcome cancer death in AMI patients. The models included the following variables: age, sex, PCI, diabetes, smoking status, platelet aggregation inhibitor at discharge and statin at discharge. Only patients who survived at least 2 years were included.

|  | **HR** | **95%-CI** | **p-value** |
| --- | --- | --- | --- |
| *Age* | 1.08 | 1.07-1.10 | <0.001 |
| *Female sex* | 0.69 | 0.52-0.90 | 0.007 |
| *Therapy: PCI* | 0.97 | 0.77-1.23 | 0.828 |
| *Diabetes* | 1.35 | 1.08-1.67 | 0.007 |
| *Smoking: currently smoking* | 2.01 | 1.58-2.55 | <0.001 |
| *never-smoker* | 0.49 | 0.36-0.66 | <0.001 |
| *Platelet aggregation inhibitor at discharge* | 0.53 | 0.31-0.91 | 0.021 |
| *Statin at discharge* | 0.83 | 0.60-1.16 | 0.280 |

Table S2: Hazard ratios (HR) and 95% confidence intervals (95%-CI) of the Cox regression analysis for the outcome lung cancer death in AMI patients. The models included the following variables: age, smoking status and education. Only patients who survived at least 2 years were included.

|  | ***HR*** | ***95%-CI*** | ***p-value*** |
| --- | --- | --- | --- |
| *age* | 1.10 | 1.07-1.13 | <0.001 |
| *Smoking: currently smoking* | 4.33 | 2.57-7.29 | <0.001 |
| *Smoking: never-smoker* | 0.13 | 0.04-0.42 | <0.001 |
| *higher educational attainment* | 0.51 | 0.28-0.92 | 0.025 |

Table S3: Hazard ratios (HR) and 95% confidence intervals (95%-CI) of the Cox regression analysis for the outcome gastrointestinal cancer death in AMI patients. The models included the following variables: age, sex, PCI, hypertension, diabetes, smoking status, platelet aggregation inhibitor at discharge and statin at discharge. Only patients who survived at least 2 years were included.

|  | ***HR*** | ***95%-CI*** | ***p-value*** |
| --- | --- | --- | --- |
| *Age* | 1.06 | 1.04-1.09 | <0.001 |
| *Female sex* | 0.66 | 0.41-1.04 | 0.076 |
| *Therapy: PCI* | 0.88 | 0.60-1.28 | 0.492 |
| *Hypertension* | 1.36 | 0.86-2.15 | 0.184 |
| *Diabetes* | 1.75 | 1.23-2.48 | 0.002 |
| *Smoking: currently smoking* | 1.39 | 0.92-2.08 | 0.114 |
| *Smoking: never-smoker* | 0.50 | 0.31-0.81 | 0.005 |
| *Platelet aggregation inhibitor at discharge* | 0.43 | 0.20-0.91 | 0.028 |
| *Statin at discharge* | 0.50 | 0.31-0.78 | 0.003 |

Table S4: Hazard ratios (HR) and 95% confidence intervals (95%-CI) of the of the Fine-Gray for the outcome cancer death in AMI patients. The models included the following variables: age, sex, PCI, diabetes, smoking status, platelet aggregation inhibitor at discharge, and statin at discharge.

|  | **HR** | **95%-CI** | **p-value** |
| --- | --- | --- | --- |
| *Age* | 1.07 | 1.05-1.08 | <0.001 |
| *Female sex* | 0.72 | 0.56-0.93 | 0.012 |
| *Therapy: PCI* | 1.00 | 0.80-1.25 | 1.000 |
| *Diabetes* | 1.17 | 0.95-1.43 | 0.140 |
| *Smoking: currently smoking* | 2.06 | 1.64-2.60 | <0.001 |
| *never-smoker* | 0.55 | 0.41-0.73 | <0.001 |
| *Platelet aggregation inhibitor at discharge* | 0.68 | 0.40-1.13 | 0.140 |
| *Statin at discharge* | 1.00 | 0.73-1.38 | 1 |

Table S5: Hazard ratios (HR) and 95% confidence intervals (95%-CI) of the Fine-Gray for the outcome lung cancer death in AMI patients. The models included the following variables: age, smoking status and education.

|  | ***HR*** | ***95%-CI*** | ***p-value*** |
| --- | --- | --- | --- |
| *age* | 1.08 | 1.06-1.11 | <0.001 |
| *Smoking: currently smoking* | 4.42 | 2.71-7.22 | <0.001 |
| *Smoking: never-smoker* | 0.16 | 0.06-0.47 | <0.001 |
| *Higher educational attainment* | 0.69 | 0.41-1.14 | 0.150 |

Table S6: Hazard ratios (HR) and 95% confidence intervals (95%-CI) of the Fine-Gray models for the outcome gastrointestinal cancer death in AMI patients. The models included the following variables: age, sex, PCI, hypertension, diabetes, smoking status, platelet aggregation inhibitor at discharge, and statin at discharge.

|  | ***HR*** | ***95%-CI*** | ***p-value*** |
| --- | --- | --- | --- |
| *Age* | 1.05 | 1.03-1.07 | <0.001 |
| *Female sex* | 0.63 | 0.39-0.99 | 0.047 |
| *Therapy: PCI* | 0.89 | 0.62-1.28 | 0.540 |
| *Hypertension* | 1.14 | 0.73-1.78 | 0.560 |
| *Diabetes* | 1.57 | 1.12-2.21 | 0.010 |
| *Smoking: currently smoking* | 1.38 | 0.93-2.05 | 0.110 |
| *Smoking: never-smoker* | 0.58 | 0.37-0.93 | 0.023 |
| *Platelet aggregation inhibitor at discharge* | 0.53 | 0.26-1.07 | 0.078 |
| *Statin at discharge* | 0.62 | 0.39-0.98 | 0.039 |

Table S7: Hazard ratios (HR) and 95% confidence intervals (95%-CI) of the Cox regression analysis for the outcome overall cancer death in AMI patients. The models included the following variables: age, sex, PCI, diabetes, smoking status, and statin at discharge. Only patients with AMI between 2010 and 2017 were included in the regression analysis.

|  | **HR** | **95%-CI** | **p-value** |
| --- | --- | --- | --- |
| *Age* | 1.09 | 1.05-1.12 | <0.001 |
| *Female sex* | 0.66 | 0.38-1.15 | 0.145 |
| *Therapy: PCI* | 0.71 | 0.43-1.17 | 0.180 |
| *Diabetes* | 1.52 | 0.97-2.39 | 0.069 |
| *Smoking: currently smoking* | 3.44 | 1.99-5.97 | <0.001 |
| *never-smoker* | 0.59 | 0.28-1.25 | 0.170 |
| *Statin at discharge* | 0.55 | 0.27-1.11 | 0.094 |

*Platelet aggregation inhibitor at discharge was omitted from the model due to a very small number of AMI patients who have not received this medication at discharge after 2010.*

**
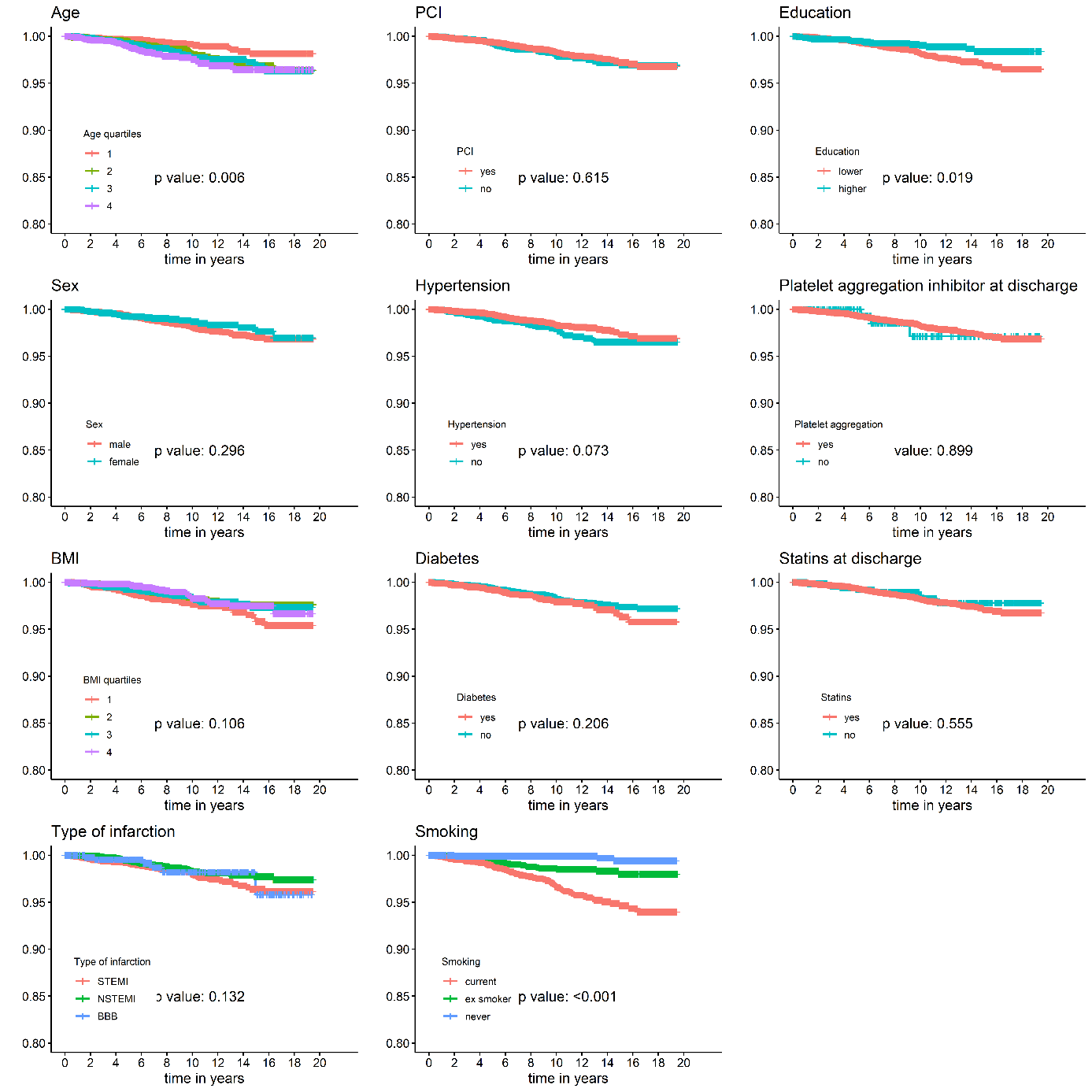
Figure S1:** Kaplan Meier curves for the event lung cancer death in AMI patients, stratified by all initially considered covariables; p-values were calculated by log-rank tests.


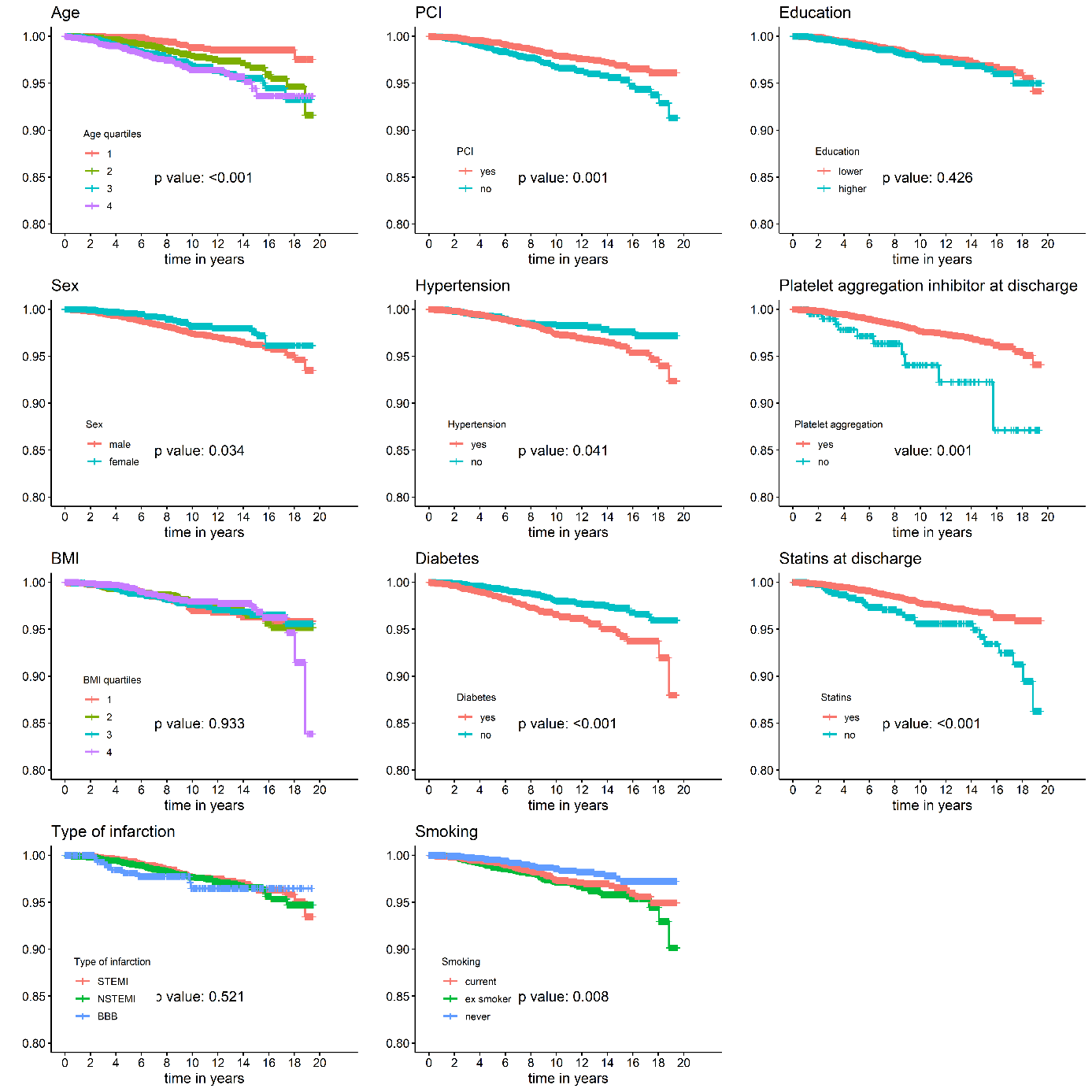


**Figure S2:** Kaplan Meier curves for the event GI cancer death in AMI patients, stratified by all initially considered covariables; p-values were calculated by log-rank tests.
